# Effects of Joint Action Observation on Children’s Imitation

**DOI:** 10.3390/bs15020208

**Published:** 2025-02-13

**Authors:** Nejra Rizvanović, Ildikó Király, Natalie Sebanz

**Affiliations:** 1 Department of Cognitive Science, Central European University, 1100 Vienna, Austria; sebanzn@ceu.edu; 2 MTA-ELTE Social Minds Research Group, Psychology Institute, Eötvös Loránd University, 1064 Budapest, Hungary; kiraly.ildiko@ppk.elte.hu

**Keywords:** social interaction, imitative learning, overimitation, joint action coordination

## Abstract

Grasping others’ intentions from their actions is essential for learning, as it enhances the ability to identify collaborative acts and anticipate others’ actions, facilitating effective coordination toward shared goals. From a young age, children seem to recognize when others are working together based on their interactions and use this understanding to inform their own learning. Although much of early learning occurs in joint contexts, little attention has been devoted to understanding how children learn by participating in joint actions and by observing others acting together. Using a puzzle box paradigm, we tested 3–6-year-old children’s imitation of an inefficient performance following individual and joint demonstrations in which the inefficient performance did or did not involve bimanual or joint coordination. This allowed us to test whether the tendency to overimitate extends to joint actions and how action coordination modulates imitative behavior. We found that overimitation extends to joint actions, as indicated by similar rates of inefficient copying following individual and joint action demonstrations. Furthermore, our results suggest that action coordination did not play a significant role in modulating children’s tendency to overimitate. Taken together, the results of the study advance our understanding of how learning occurs in social interactions.

## 1. Introduction

Young children routinely incorporate actions which are causally irrelevant, inefficient, or somehow ‘silly’ into their imitation (Hoehl et al., 2019; Horner & Whiten, 2005; Lyons et al., 2007). Such high-fidelity copying is an early-emerging form of social learning, appearing between the ages of 18 months and 3 years (Gardiner et al., 2011; Whiten et al., 2009), increasing into childhood (Horner & Whiten, 2005; Lyons et al., 2007; McGuigan et al., 2007) and adulthood (McGuigan et al., 2011; Flynn & Smith, 2012). Unlike emulation, which prioritizes reproducing the observable, concrete goal without replicating the specific means, imitation entails a full reproduction of the modeled action. This includes both the action’s form—i.e., how it was achieved—and its intended outcome—i.e., what was achieved (Csibra & Gergely, 2006; Whiten et al., 2009). When the modeled action includes causally irrelevant elements (e.g., actions that do not contribute to achieving the end-goal), this behavior is referred to as ‘overimitation’ (Lyons et al., 2007).

Theorists of cultural evolution have argued that in addition to other social learning mechanisms such as emulation and teaching, the human predilection towards high-fidelity copying may form the bedrock of culture. In fact, as a nonselective copying strategy, it enables the rapid acquisition of causally opaque forms of knowledge (i.e., behaviors which lack an obvious instrumental function), such as cultural conventions and rituals, that are readily available during childhood but difficult to acquire through individual practice (Boyd et al., 2011; Kapitány & Nielsen, 2015; Lyons et al., 2011; Nielsen & Tomaselli, 2010).

Although overimitation has been studied extensively in contexts where child learners observe and learn novel actions from individual demonstrations, much less is known about how observing and participating in joint actions guides learning (Charbonneau et al., 2024; McEllin et al., 2018). The current research, therefore, aimed to fill this gap by investigating how preschool children interpret unusual, causally irrelevant actions performed in a joint context, and how they imitate them when doing so entails coordination with another person. Specifically, we investigated whether the tendency to overimitate, which has been documented for individual actions (Lyons et al., 2007), extends to joint actions and whether joint coordination modulates imitative behavior. By exploring these questions, we sought to deepen our understanding of the role of social interaction in early learning and contribute to a growing body of work that is demonstrating children’s imitative flexibility (Legare et al., 2015). From a practical perspective, insights from this work could inform the design of targeted interventions that approach learning as a form of joint, collaborative activity.

Below, we first summarize key findings concerning the processes and cognitive mechanisms that support flexible imitative learning from individual action observation in childhood and outline the ways in which these may be applied to learning about and from joint actions. Next, we review research on the development of joint action and suggest how the key elements that are involved in understanding and participating in joint actions may inform imitation of joint actions.

### 1.1. Processes Underlying Flexible Imitation of Actions

Although children have a strong tendency to imitate others faithfully, research reveals that they are highly selective imitators who strategically decide when to replicate an outcome (e.g., emulate) and when to replicate the process (e.g., imitate). Indeed, they are able to generate different predictions about observed behaviors based on what they perceive to be the model’s goal during the demonstration (Clegg & Legare, 2016; Gergely et al., 2002; Gergely & Jacob, 2012; Király et al., 2013; Over & Carpenter, 2009; Rakoczy & Schmidt, 2013) and adjust their imitative strategies depending on whether they are learning instrumental skills or social and normative conventions (Clegg & Legare, 2016; Rakoczy & Schmidt, 2013). We argue that it is precisely the flexible nature of imitation, subserved by humans’ precocious ability to reason about others’ actions in terms of goals (Csibra, 2003), that allows children to understand and learn from various forms of social interaction, such as joint actions—for instance, by enabling them to go beyond reasoning about individual goals—and to learn from and about the complementary nature of coordinated, joint actions (Brownell, 2011; Henderson & Woodward, 2011; Warneken et al., 2006; Warneken & Tomasello, 2007).

Proponents of the *affiliative account* suggest that overimitation occurs because of a deeply engrained social motivation to belong to a social group, expressed in the imitative context through the desire to affiliate with and act like the model (Over & Carpenter, 2013; Over, 2020). In an experimental demonstration of this by Nielsen and Blank (2011), children’s imitative strategy depended on which demonstrator was physically present in the room at the time the child manipulated the puzzle box. In this study, children aged 4–5 years observed two methods of opening a puzzle box—one efficient and the other inefficient. While they were more likely to adopt the inefficient technique if the model demonstrating it was present during imitation, when left alone, they tended to copy the efficient method more (Nielsen & Blank, 2011). Findings from this study suggest that merely invoking a social scenario (e.g., by having the model that demonstrated the action present in the room) leads children to copy that model’s course of action despite their knowledge of the efficient strategy. In other words, when the learning context emphasized social goals, and the children felt a strong motivation to affiliate with the model, they often imitated the person, and not the actions.

Crucially, the ability and motivation to faithfully imitate a model’s actions are considered fundamental for learning group-specific cultural conventions, such as norms and rituals (Heyes, 2021; Nielsen et al., 2020), that promote group coordination and cooperation (Henrich, 2009; Legare & Nielsen, 2020; Whitehouse, 2021) by signaling group membership (Liberman et al., 2018), in-group preferences (Wen et al., 2016), and fostering group trust (Hobson et al., 2018).

Relatedly, proponents of the normative account suggest that overimitation may result from children interpreting the demonstrated actions as social norms (Hoehl et al., 2019; Keupp et al., 2013). According to this view, children’s representation of events is organized in a hierarchical goal-like structure, which renders a certain goal more important in the action parsing process depending on contextual cues, resulting in a flexible use/occurrence of overimitation (Keupp et al., 2013). For instance, when children determine that bringing about the effect is hierarchically the most important goal, they should choose a course of action suitable for reaching that goal without necessarily copying the same means produced by the model. If, however, they consider that copying the same means produced by the model is the most important goal, they should imitate the means in addition to the end-goal.

Recent findings support the view of overimitation as a function of normative action interpretation. For instance, studies show that starting at around 2 years of age, children can form different representations depending on the context in which the action was produced (Keupp et al., 2013, 2015; Over & Carpenter, 2009; Rakoczy & Schmidt, 2013; Watson-Jones et al., 2014) and tend to imitate causally irrelevant actions more in conventional compared to instrumental contexts (Clegg & Legare, 2016; Herrmann et al., 2013; Kenward et al., 2011; Kenward, 2012; Rakoczy & Schmidt, 2013; Watson-Jones et al., 2014). When normative language was used to highlight that an inefficient demonstration has a social function and represents norms and conventions, 3–6-year-old children were more likely to copy causally unnecessary actions compared to when such actions were marked in non-normative, instrumental terms (Kenward, 2012; Watson-Jones et al., 2014). A possible reason for why children considered that reproducing the entire action sequence is the most important goal despite being able to discern the causal structure of the task is that the presence of the model and the use of normative language led the children to encode the causally irrelevant actions as socially/normatively rather than instrumentally relevant.

Recent research in developmental psychology with 9- to 14-month-old infants suggests that observers also use information about *action efficiency* in a flexible way to support their understanding of collaborative and cooperative behaviors (Begus et al., 2020; Mascaro & Csibra, 2022; Vizmathy et al., 2024). These build on prior work demonstrating infants’ capacity for representing the goals of both individual (e.g., Gergely & Csibra, 2003; Hernik & Southgate, 2012; Woodward, 1998) and collaborative actions (Fawcett & Liszkowski, 2012; Henderson & Woodward, 2011; Henderson et al., 2013; Krogh-Jespersen et al., 2020).

Indeed, whereas evidence shows that at 12 months, infants expect agents to act according to the principle of rationality and be as efficient as possible when pursuing *individual* goals (e.g., Csibra, 2003; Gergely et al., 2002; Jara-Ettinger et al., 2016; Liu et al., 2019; Scott & Baillargeon, 2013; Skerry et al., 2013), these expectations of efficiency are suspended in a joint context where two agents are coordinating to reach a *shared goal* (Begus et al., 2020; Vizmathy et al., 2024). A possible explanation for this finding comes from research on ‘sensorimotor’ or movement-based communication in joint action research with adults, showing that inefficient behaviors in the form of movement modulations (e.g., speeding up, slowing down, or exaggerating one’s movement trajectory) often serve to facilitate coordination in a joint context by increasing the predictability of partners’ actions (Pezzulo et al., 2013; Vesper et al., 2011; Vesper & Richardson, 2014). Similarly, research has shown that when engaged in joint actions, adults routinely prioritize joint efficiency over individual efficiency by choosing paths that minimize joint rather than individual costs (Török et al., 2019, 2021). Given the facilitating role of sensorimotor communication in joint actions and the demonstrated willingness of joint action partners to forgo individual efficiency when coordinating, deviations in efficiency that are often a result of such signaling may be justified in the context when coordinating with another is necessary to reach the intended goal. In fact, to observers, such deviations from efficiency may serve to support the understanding of collaborative actions (e.g., Begus et al., 2020; Vizmathy et al., 2024).

It remains an open question, however, whether observing jointly performed causally irrelevant, and therefore inefficient, actions would lead to faithful *copying*—perhaps due to suspending individual efficiency expectations when there is a need to coordinate with a partner (e.g., Begus et al., 2020; Vizmathy et al., 2024) or interpreting such actions as socially rather than instrumentally relevant (Clegg & Legare, 2016; Kenward et al., 2011; Kenward, 2012; Keupp et al., 2013; Legare et al., 2015). Investigating how observers *imitate* jointly performed inefficient actions could provide further insight into the way they reason about action inefficiency in a joint context—where inefficiency is achieved by performing non-functional, causally irrelevant actions. Additionally, it can specify how the immediate need to coordinate with a partner guides imitative learning and, in this way, offer a more nuanced understanding of the way joint activities influence and shape the transmission of cultural information in childhood.

### 1.2. Imitation of Coordinated Actions

In imitative learning contexts, interpersonal coordination has also been linked to higher fidelity in childhood. For instance, in a study by Herrmann et al. (2013), 3–6-year-old children observing coordinated actions performed by two models in parallel copied such actions more faithfully compared to asynchronous actions demonstrated twice by a single individual or once by two models in succession. The authors concluded that observing multiple individuals performing the same action in parallel prompted children to interpret the observed action in conventional rather than instrumental terms—that is, children interpreted the actions performed by multiple individuals as reflecting conventional knowledge rather than knowledge about how to perform the action in an instrumentally efficient way (Herrmann et al., 2013).

Following this rationale, it can be argued that, to the extent that joint actions are considered to represent culturally shared information because they are brought about through shared intentionality (Bratman, 1992), they should be imitated with greater fidelity compared to actions that are brought about individually. In this way, reasoning about joint actions may be akin to reasoning about social norms and conventions (Rakoczy & Schmidt, 2013).

Although the study by Herrmann et al. (2013) sheds light on the role of interpersonal coordination on imitative fidelity, it is not clear whether children in this study conceived of the coordinated behavior they saw as joint, as the two models performed actions on separate objects in parallel. A more recent study by Milward and Sebanz (2018) explored the effects of joint action observation and participation on the imitation of closely coordinated actions in 2.5–6-year-old children. Children either observed two actors performing two different parts of a joint action or participated in the joint action themselves. The authors found that the children were more likely to replicate both parts of the joint action following joint action observation compared to participation. These findings provide the first direct evidence about children’s imitation of *coordinated* actions and suggest that it may be easier for children at this stage in development to form joint goal representations when they passively observe a joint action compared to when they are actively involved in the task. Similarly, an investigation into the effects of joint action observation on imitation by Fawcett and Liszkowski (2012) showed that 18-month-old children are more likely to recruit a partner and replicate the joint activity after having observed joint actions compared to observing parallel or individual actions.

Overall, this early sensitivity suggests an existing set of cognitive mechanisms that allows observers to go beyond the analysis of individual actions and learn from and about the complementary nature of joint actions (Henderson & Woodward, 2011; Warneken et al., 2006; Warneken & Tomasello, 2007). Furthermore, it suggests that joint actions may be important in the stabilization of cultural traditions by boosting high-fidelity copying for individuals that observe (Milward & Sebanz, 2018) and partake (Fawcett & Liszkowski, 2012) in joint activities.

Although the studies above show that children can recognize joint goals by observing others’ coordinated behavior (Milward & Sebanz, 2018) and tend to replicate coordinated behaviors jointly (Fawcett & Liszkowski, 2012), it remains an open question whether observing jointly coordinated behavior also creates an expectation that such actions should be imitated faithfully, even if they are not functional in bringing about the desired shared goal (i.e., if they include causally irrelevant actions)? Exploring this question could offer deeper insight into the role of joint actions in stabilizing cultural traditions and enhance our understanding of how children interpret and make sense of others’ actions in social and collaborative contexts.

### 1.3. Purpose of the Present Study

In the current study, we examined whether the tendency of young children to copy causally irrelevant actions (i.e., to overimitate) following individual demonstrations (e.g., Lyons et al., 2007) extends to joint demonstrations. Furthermore, we investigated whether observing joint behavior, in which two individuals perform causally irrelevant actions while coordinating to achieve a shared instrumental goal, leads to greater imitation of these actions compared to their performance in an individual, non-coordinated context. We based our design on previous studies on overimitation that utilized puzzle boxes with embedded causally irrelevant actions to examine how children interpret and imitate these actions under different conditions (Hoehl et al., 2019). However, we modified the apparatus to allow joint manipulation (see Section 2.2 for details on the design of the task apparatus). While prior studies have incorporated certain elements of joint actions into their designs (e.g., Begus et al., 2020; Herrmann et al., 2013; Milward & Sebanz, 2018), to our knowledge, no study to date has directly investigated the link between joint action observation and overimitation by embedding goal-irrelevant elements into the task, nor has one aimed to disentangle the relative contributions of joint goals and action coordination within an instrumental, goal-directed task.

To that end, we employed a 2 × 2 between-subject design, experimentally varying two factors: the type of action children observed (individual vs. joint) and the presence of action coordination involved in the performance of the causally irrelevant action (present/yes vs. absent/no). This resulted in four experimental conditions, to which the children were assigned randomly: individual coordination, individual no coordination, joint coordination, and joint no coordination.

We predicted that observing joint coordinated actions would result in more imitation of the causally irrelevant action compared to observing these actions when performed in an individual, non-coordinated context. This may be due to suspending individual efficiency expectations when there is a need to coordinate with another person (e.g., Begus et al., 2020; Vizmathy et al., 2024) or because of a stronger bias for interpreting causally irrelevant actions as socially relevant in a joint context—i.e., relevant for the social goal of acting together—rather than instrumentally relevant—i.e., relevant with respect to the instrumental outcome of the task (Clegg & Legare, 2016; Kenward et al., 2011; Kenward, 2012; Keupp et al., 2013; Legare et al., 2015; Moraru et al., 2016). It is important to note that although a normative bias begins to emerge early on for *individual* actions if they are marked in conventional (Clegg & Legare, 2016; Kenward et al., 2011; Kenward, 2012; Legare et al., 2015; Rakoczy & Schmidt, 2013; Watson-Jones et al., 2014) terms, observing *joint* actions may elicit a stronger normative bias because normativity is further emphasized via coordinated action. Thus, if participants are more likely to perceive a performance as normative because it is produced in a joint context (e.g., where cues of conventionality are pronounced by shared goals and joint coordination), they should imitate the causally irrelevant action more in a joint compared to an individual context.

## 2. Materials and Methods

### 2.1. Participants

A total of 101 children aged 3 to 6 years (*M* = 4.7 years, *SD* = 0.829, 49 females) that participated in the study were included in the final analysis. This age range was selected because it marks a developmental period when children can form joint goal representations (Warneken et al., 2012) and when their ability to reason about actions in normative terms matures, driven in part by prolonged exposure to social norms and conventions (Rakoczy & Schmidt, 2013). Several local public kindergartens were contacted via email or in person, and parental consent forms were distributed to those who expressed interest in participating. All children whose parents provided prior consent were tested at their respective kindergartens. Participants were typically developing speakers of German or Hungarian. No additional selection criteria were applied beyond the age range and the ability to understand German or Hungarian.

We based our sample size on a previous imitation study that used a comparable number of participants per condition and tested a similar age range (Schleihauf et al., 2019). The sample size in the study by Schleihauf et al. (2019) was determined through an a priori power analysis conducted using G∗Power (version 3.1.9.2; Faul et al., 2009). The analysis was informed by prior research on overimitation (Hoehl et al., 2014), with an expected effect size of d ≥ 0.6, a significance level of *p* < 0.05 (two-tailed), and a statistical power of 1 − β = 0.8.

The study included four experimental between-subject conditions: individual coordination (n = 25), individual no coordination (n = 25), joint coordination (n = 26), and joint no coordination (n = 25). The final sample comprised 7 three-year-olds, 32 four-year-olds, 45 five-year-olds, and 17 six-year-olds. Thirteen additional participants were recruited but excluded from the final sample because they did not complete the testing either due to shyness (n = 7), restlessness (n = 1), or experimenter error (n = 5). Participants were tested in kindergartens in Vienna, Austria (n = 42, females = 22) and in Budapest, Hungary (n = 59, females = 27). Upon completing the study, all children received stickers and a participation certificate as recompense, irrespective of their success at the task. The experiment was approved by the Psychological Research Ethics Board (PREBO) in Austria (approval code: 2021/08_01) and the United Ethical Review Committee for Research in Psychology (EPKEB) in Hungary (approval code: 2022-129). We collected written consent from children’s parents or primary caretakers. In addition, participants provided verbal consent before taking part in the experiment and signed their name on the consent form either alone or with the help of the experimenter.

All sessions were video recorded with the permission of participants’ parents/primary caretakers and coded on the relevant measures. The data were analyzed using the 0.16.4.0 version of the JASP data analysis and visualization package.

### 2.2. Materials and Task

**Figure 1:**
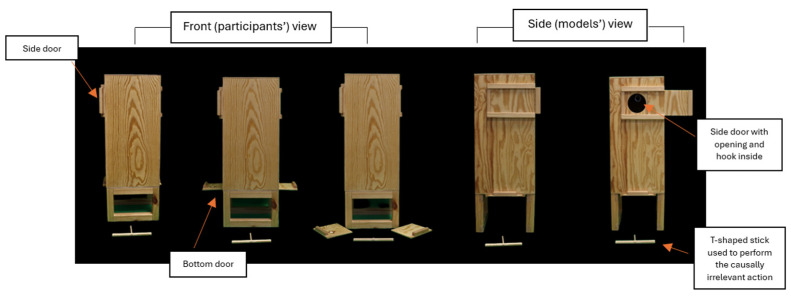
Illustration of the apparatus depicting its integral features, as well as the wooden stick used to perform the causally irrelevant action (CIA). The integral features include a top sliding door, behind which there is an opening (one on each side of the box), and a bottom sliding door, atop which is an opening (spanning both sides of the box).

A 30 × 70 × 30 cm opaque (wooden) puzzle box was used across conditions (see Figure 1 for an illustration). The apparatus was constructed specifically for this study, drawing on designs commonly used in imitation research that examine children’s understanding and imitation of objects (e.g., Hoehl et al., 2014; Horner & Whiten, 2005; Lyons et al., 2007; Schleihauf et al., 2021). The primary distinction was that, while such apparatuses are typically designed for manipulation by a single individual only, ours was modified to enable additional manipulation by two individuals (i.e., joint action) by incorporating mirrored features.

A hidden object with a sticker inside could be retrieved from the puzzle box by performing a sequence of causally relevant actions in which the performance of a single causally irrelevant action was embedded. The causally relevant actions included (a) opening the top sliding doors, (b) detaching the hooks from the loops inside the puzzle box, and (c) opening the bottom sliding doors. Since the box features were mirrored on both of its sides, there were a total of six causally relevant actions (three on each side). Opening the top sliding doors revealed the loops inside the box, from which the hidden object was suspended by a string. Detaching the hooks caused the object to drop to the bottom of the box, which was hollow but secured by the bottom sliding doors. Once these doors were opened and removed completely, the object fell out of the box and could be retrieved. The causally irrelevant action consisted in moving a T-shaped stick (illustrated below) to touch the top front of the box. All causally relevant actions were performed in the same way by the model(s), while we experimentally varied the presence of individual or joint action coordination when performing the causally irrelevant action.

### 2.3. Design

**Figure 2:**
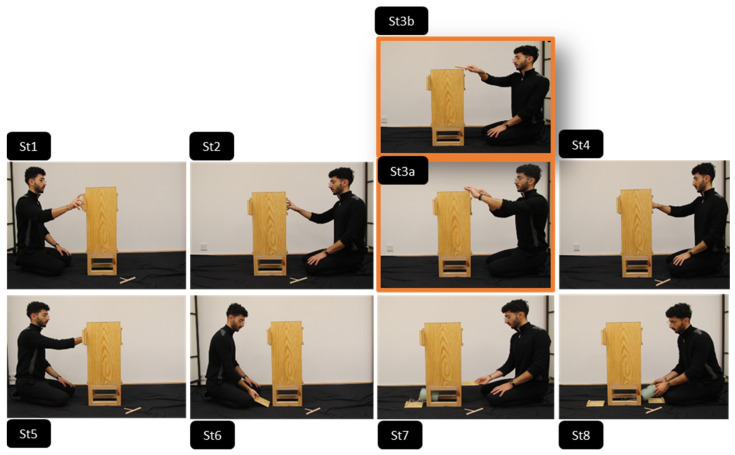
Illustration of the individual demonstration condition and actions performed by the model. The model retrieved the hidden object from the puzzle box by (**a**) opening the top sliding doors (St1, St2); (**b**) grabbing the T-shaped stick and moving it to tap the top of the box either bimanually/i.e., with coordination (St3a, in orange) or unimanually/i.e., without coordination (St3b, in orange); (**c**) reaching into a round opening to remove the hook that held the object suspended on a loop inside the box (St4, St5); (**d**) opening the bottom sliding doors which revealed the previously hidden object (St6, St7); and (**e**) retrieving the object (St8).

**Figure 3:**
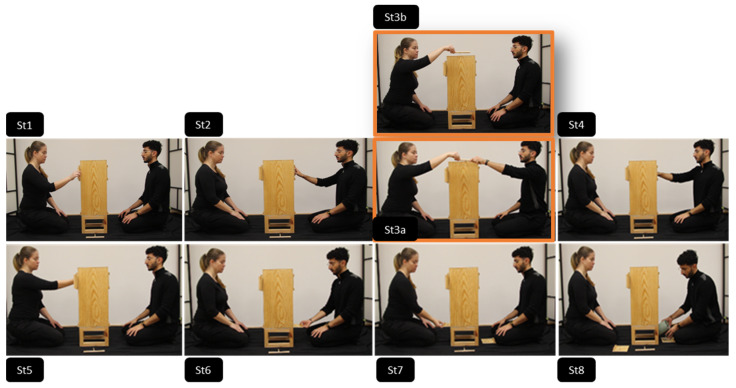
Illustration of the joint demonstration condition and actions performed by the model. The models retrieved the hidden object from the puzzle box by taking turns in (**a**) opening the top sliding doors (St1, St2); (**b**) grabbing the T-shaped stick with one hand and moving it to tap the top of the box either together/joint coordination (St3a, in orange) or not/no coordination (St3b, in orange); (**c**) reaching into a round opening to remove the hook that held the object suspended on a loop inside the box (St4, St5); (**d**) opening the bottom sliding doors which revealed the previously hidden object (St6, St7); and (**e**) retrieving the object (St8). A single stick was always used during the demonstration, meaning that in the joint coordination condition, both demonstrators manipulated the same stick using one hand each (i.e., jointly).

Participants were randomly assigned to one of the four conditions described in detail in Table 1. See also Figure 2 and Figure 3 for a visual representation.

Participants in the individual observation group watched a single model performing the causally irrelevant action either unimanually (no coordination condition) or bimanually (coordination condition). In contrast, participants in the joint observation group watched a dyad, where either one member performed the action unimanually (no coordination condition) or both members manipulated the stick together using one hand each (joint coordination) (See Figure 2 and Figure 3).

In the individual observation conditions, a single model performed all actions alone, moving from one side of the box to the other. This included performing all six causally relevant actions, as well as the causally irrelevant action that was embedded within the action sequence (marked in orange in Figure 2). In contrast, in the joint condition, the two members of the dyad alternated manipulating their side of the box while seated, each performing three causally relevant actions. Depending on the condition, the causally irrelevant action was performed either by one member unimanually or by both members simultaneously (marked in orange in Figure 3). Full videos of the demonstrations can be found in the Supplementary Materials at https://osf.io/hsg2n/ (accessed on 9 February 2025).

This design ensured that the demonstration of the causally irrelevant action was kept perceptually consistent across conditions (i.e., always performed with one or two hands; see Table 1 for an overview), allowing any differences in overimitation rates between conditions to be attributed to the social context in which the action was performed.

### 2.4. Procedure

Prior to taking part in the study, participants provided verbal consent and proceeded to sign the consent form either on their own or with the help of the experimenter. Then, they were encouraged to explore the puzzle box on their own for a maximum of two minutes. After the child signaled that they were finished exploring the box or after the two minutes had elapsed, they were led to and seated in a chair which was positioned centrally at 1.5 m facing the box. Participants then observed the model(s) demonstrating how to open the puzzle box to retrieve the hidden object, after which they were invited to operate the box themselves, either alone (individual action condition) or together with one of the adult models (joint action condition). Each trial thus included an exploration phase, a demonstration phase, and a test phase.

*Exploration phase.* Participants were brought to the testing room individually and were encouraged to explore the object on their own, with the models present throughout the phase but immersed in another activity or idle. The T-shaped stick was placed next to the box and could thus be explored by children. However, the reward box inside the apparatus was removed to prevent children from discovering the purpose of the demonstration before having a chance to see it modeled by the experimenter. While one of the models remained seated at a desk and pretended to write on a piece of paper, another stood next to her, looking in her direction and away from the child. However, the idle model was encouraging if they saw that the child was too shy to explore the box or otherwise insecure about what to do. If asked, they refrained from answering specific questions about how the box works but gave general directions and words of encouragement to the child to “go ahead and try it out” and to explore the box from all sides.

*Demonstration phase*. To begin the demonstration phase of the individual condition, Model 1 began by addressing the child, “Now **I** am going to show you how to open the box and find something that’s hidden inside”, and retrieved the hidden object from the puzzle box, saying “Oh, look!” and looking at the child. In the joint condition, Model 1 first invited Model 2 (who was engaged in a different task and sitting at a desk away from Model 1 and the child) to join the activity and said, “Will you do this with me?”. Model 2 agreed and sat on the opposite side of the box. Model 1 then addressed the child, saying “Now **we** are going to show you how to open the box and find something that’s hidden inside”, and retrieved the hidden object from the puzzle box, saying “Oh, look!” and taking turns looking at the child and at Model 2. The child was then escorted out of the room by one experimenter while the other reassembled the box.

*Test phase.* In the individual condition, the test phase began when Model 1 turned to the child and said “It’s your turn now”, moving away from their starting position and away from the puzzle box.^1^ Regardless of the condition, the action sequence was always initiated by the model sitting on the left-hand side (i.e., Model 2 in the joint condition), which was also the side the participant occupied when acting on the box. In the joint action condition, after exclaiming “It’s your turn now”, Model 1 remained in their position, sitting idly next to the box, and waited for the child to initiate the action sequence. Model 2 returned to their initial position at the desk and immersed themselves in another activity (e.g., writing on a piece of paper). After the child initiated the action sequence, Model 1 followed closely with the same action (i.e., if the child started the sequence performing the first causally relevant action, Model 1 performed the same causally relevant action on their side of the box). Importantly, Model 1 always waited to see if the child will reach for the stick (and grab it on one side only) in order to perform the causally irrelevant action before mirroring the child’s actions and performing the action with them (joint coordination). In the no coordination condition, Model 1 would refrain from performing the causally irrelevant action unless invited by the child either verbally or non-verbally (e.g., staring, waiting for the Model to join in, and/or pointing). After retrieving the hidden object, the child could choose a sticker and was given a certificate of participation as a token of appreciation for taking part in the study.

### 2.5. Coding and Reliability

*Replication of the causally irrelevant action.* We considered any behaviors aimed at reproducing the irrelevant action as replications. These included reaching movements to grasp the T-shaped stick in the same or different manner as the one demonstrated by the model(s) (e.g., by using a different hand or one hand instead of two) and using it to touch the front top of the puzzle box. As this was a binary outcome measure indicating the presence or absence of overimitation, we calculated the score in the following manner: 0 = no replication, 1 = replication of the causally irrelevant action.

The coding criteria were a result of expert consensus and based on prior research that employed a similar approach to assessing overimitation by evaluating the presence or absence of imitated steps and/or the presence or absence of related features of interest (for different examples, see Kenward et al., 2011; Keupp et al., 2013; Legare et al., 2015; Schleihauf et al., 2019).

Children’s responses were coded from the videotapes by the first author. To assess inter-rater reliability, a second coder—who was one of the experimenters that was trained on the agreed-upon criteria, but blind to the study’s hypotheses as well as the condition to which each child was assigned—re-coded 25 videos (approximately 25% of the dataset). The Kappa coefficient was calculated and indicated perfect agreement (*κ* = 1) between the two coders regarding whether the causally irrelevant action was imitated.

## 3. Results

### Replication of the Causally Irrelevant Action

To examine whether children were more likely to imitate the causally irrelevant action after observing a joint demonstration, a logistic regression analysis was conducted for binary data. Social condition (individual vs. joint) and action coordination (yes vs. no) were included as between-subject factors, with overimitation as the dependent variable, with two possible response values of 0 (indicating absence of overimitation), and 1 (indicating presence of overimitation). The above-described between-subject study design with binary outcome data justified the choice of logistic regression as the main method of analysis.

The results of the logistic regression analysis indicated that the odds of reproducing the causally irrelevant action were 1.44 times higher following individual demonstrations (58% imitated) compared to joint demonstrations (49% imitated). However, this difference was not statistically significant, 95% CI [−0.42, 1.15], *p* = 0.367. The corresponding effect size, Cohen’s *d* = 0.20, suggests a small effect. Similarly, there was no statistically significant difference in the odds of reproducing the causally irrelevant action between coordinated demonstrations (53% imitated) and non-coordinated demonstrations (54% imitated). Participants in the non-coordination conditions were 1.04 times as likely to reproduce the causally irrelevant action as those in the coordination conditions, 95% CI [−0.75, 0.83], *p* = 0.922 (see Table 2). The corresponding effect size, Cohen’s *d* = 0.02, indicates a negligible effect.

The interaction between social condition and action coordination revealed that when the demonstration was not coordinated; the odds of reproducing the causally irrelevant action were 2.26 times higher following individual demonstrations (64% imitated) compared to joint demonstrations (44% imitated). However, this difference was not statistically significant, 95% CI [−0.32, 1.95], *p* = 0.159. The corresponding effect size, Cohen’s *d* = 0.45, suggests a medium effect. Conversely, when coordination was present, there was no significant difference in the odds of reproducing the causally irrelevant action between individual demonstrations (52% imitated) and joint demonstrations (54% imitated). Participants in the joint coordinated condition were 1.08 times as likely to reproduce the causally irrelevant action as those in the individual coordinated condition, 95% CI [−1.03, 1.17], *p* = 0.895. The corresponding effect size, Cohen’s *d* = 0.04, indicates a negligible effect. 

Additional exploratory analyses on age and overimitation are presented and discussed in the Appendix A.

## 4. Discussion

In the present study, we sought to understand the impact of joint actions on the imitative behavior of preschool children. To that end, we asked whether 3–6-year-olds are more likely to replicate causally irrelevant actions following joint compared to individual demonstrations of an instrumental (i.e., goal-directed) action sequence. We hypothesized that the tendency to overimitate extends to joint actions. Specifically, we predicted that compared to observing causally irrelevant actions in an individual context, observing such actions in a joint coordinated context would evoke a stronger socio-normative interpretation of the demonstrated behavior (e.g., Kenward et al., 2011; Kenward, 2012; Keupp et al., 2013) and lead children to suspend their expectations of individual efficiency (Begus et al., 2020; Vizmathy et al., 2024). This, in turn, would lead to higher rates of overimitation when reproducing the behavior with a partner.

Results from our study revealed that overimitation extends to joint actions, indicated by equal rates of copying the causally irrelevant action following both individual and joint demonstrations. However, our analyses did not show any statistically significant differences in the expected direction—that is, children were not more likely to overimitate after joint demonstrations. In addition, action coordination did not seem to modulate children’s imitative behavior. Instead, children were equally likely to copy the causally irrelevant action after individual and joint demonstrations, regardless of the presence or absence of action coordination involved in its performance.

### 4.1. Study Limitations

Several factors may explain the absence of a significant difference in imitation rates between individual and joint action conditions. First, we suspect that a lack of interactive cues between the two models performing jointly may have contributed to the lower rates of overimitation in this condition. Indeed, the two models only briefly interacted before the demonstration began, with Model 1 inviting Model 2 to participate in the demonstration (see Section 2.4). However, during the demonstration phase, the models were instructed to avoid looking at each other. This was performed to ensure that the joint action condition closely resembled the individual action condition, where a single agent operated the box without the possibility of interaction. Additionally, it aimed to rule out the influence of ostension during the joint performance of the causally irrelevant action, which has been shown to elicit overimitation (Csibra & Gergely, 2011), thus ensuring that any increase in overimitation would be attributed to the social context in which the action occurred. In hindsight, we suspect that such minimal interaction may have been insufficient in helping children disambiguate the event as joint. Indeed, based on the work of Fawcett and Gredebäck (2013), 18-month-old children were able to bind two agents’ actions into a collaborative goal only when they observed them interacting socially before *and* during the demonstration of a novel action sequence. In addition, given that ostensive cues, such as eye gaze, are important for signaling commitment to a cooperative goal in young children during individual demonstrations (Siposova et al., 2018), the absence of such cues between models performing jointly may have further hindered children’s ability to interpret the event as a cooperative, joint action.

Relatedly, it is important to note that our task was designed so that, in the joint conditions, only one of the models retrieved the object from the puzzle box at the end of the demonstration. Such a setup may have indeed made it more difficult for children to infer a joint goal, potentially leading them to construe of the event as an act of helping in which one model assisted in achieving another’s individual goal, rather than as a joint action in which both models worked towards a shared outcome. Consequently, this could have influenced children’s behavior during joint action; for example, children may have imitated the goal of a single model rather than the shared goal, resulting in lower rates of overimitation. Therefore, future work investigating the influence of joint goals on imitative fidelity should make the joint goal more salient—for example, by having the models share rewards at the end, make eye contact, or interact socially in different ways with one another throughout the demonstration.

While previous observational studies with infants showed that a turn-taking sequence helps infants encode joint goals because it allows them to infer the causal relationship between co-actors’ actions (Henderson & Woodward, 2011), the findings of this study suggest that synchronous coordination, as a cue to conventionality, may be necessary in joint imitation contexts to elicit overimitation (e.g., Herrmann et al., 2013). Nevertheless, future studies could disambiguate the relative influence of action synchronicity and shared goals on imitative fidelity by having models share rewards at the end while also acting synchronously *throughout* the joint demonstration—rather than doing so only while performing the causally irrelevant action.

Another possibility is that our method of assessing children’s motivation to overimitate was too stringent. Specifically, the model acting with the child in joint conditions was instructed to wait for the child to initiate both the overall action sequence as well as the causally irrelevant action. This design aimed to mirror Fawcett and Liszkowski’s (2012) approach, which tested whether children are more likely to initiate joint actions after observing a joint demonstration. However, while the relevant actions in the current study were performed in a turn-taking manner, the causally irrelevant action was carried out synchronously. As a result, the model could prompt the child to begin by performing the first relevant step if they were unsure about what to do (and actively mirrored them by performing the second relevant step) but could only passively wait for the child to initiate the performance of the causally irrelevant action or at least to confidently signal their intention to do so. This design aimed to ensure that the child’s intention to perform the action was self-driven and not influenced by the model. However, the model’s passive behavior may have been misinterpreted by children as hesitation, potentially discouraging them from attempting to replicate the causally irrelevant action even if they had intended to do so. Additional features absent in Fawcett and Liszkowski (2012) are the large size of the puzzle box, and its central positioning obstructing a clear view of the partner may have caused additional difficulties in signaling an existing intention to overimitate. Thus, future research could heighten model responsiveness or encourage children to verbalize their thoughts while imitating. The latter, in particular, could offer deeper insights into children’s underlying thought processes during replication.

Moreover, it is possible that causally irrelevant actions are perceived differently in instrumental versus ritualistic contexts, irrespective of the social context in which they occur. Specifically, the presence of an instrumental goal in our study (e.g., opening the box to retrieve a reward) may have led to lower rates of overimitation, as it provided information about the event’s causal structure. This, in turn, may have been used to evaluate the function and relevance of individual action steps, including the causally irrelevant action (Bauer & Mandler, 1989; in Király, 2009). In contrast, ritualistic actions, which are typically oriented toward non-instrumental outcomes—such as following specific rules, like performing a particular action sequence during a task (e.g., Herrmann et al., 2013) or arranging tokens in a specific pattern before placing them in a tube to earn stickers (Zhao et al., 2024, with findings in 4- to 6-year-olds)—may emphasize the socionormative nature of the task, as no instrumental goal is available against which to evaluate the relevance of the individual action elements. Indeed, behaviors lacking an apparent instrumental purpose are more likely to be perceived as cultural conventions and imitated more faithfully by preschoolers (Herrmann et al., 2013). In such situations, the behavioral means of the models are often interpreted as the intended goal itself (e.g., Carpenter et al., 2005).

### 4.2. Conclusions and Future Directions

Overall, the results of this study suggest that the phenomenon of overimitation that has been documented for individual actions (Lyons et al., 2007) extends to joint actions and, as such, showcases the importance of studying imitative learning in a wider range of social contexts.

Although we found no evidence that the joint context enhances overimitation in 3–6-year-old children, we suppose that the lack of difference could be due in part to overimitation already being highly social, even in individual settings (Over & Carpenter, 2013). In other words, learners may imitate causally irrelevant actions in individual contexts not only to learn important skills but also to express affiliation with the model and satisfy their need for social belonging (Over & Carpenter, 2013). Thus, the presence of a collaborative partner may not be essential to elicit high-fidelity copying.

Additionally, pedagogical features present in our design that are a general characteristic of imitative learning contexts (Hoehl et al., 2019) may have prompted children to imitate at high rates even in individual action conditions, thereby obscuring any differences that could arise from the different social contexts. In these scenarios, models typically make eye contact with child participants before the demonstration, address them by name, and direct attention to a new and exciting task that the child is meant to learn (Hoehl et al., 2019). Such a setup is likely to create an expectation in the child that the information being presented is specifically intended for them and represents new, relevant, and widely shared knowledge worth imitating (Csibra & Gergely, 2009; Gergely & Jacob, 2012; Gergely & Király, 2024; Király et al., 2013). Similarly, children may have been driven to overimitate at high rates due to audience effects (e.g., Marsh et al., 2019; Nielsen & Blank, 2011), regardless of the social context in which the action was observed. In other words, the mere presence of the model as an audience may have been sufficient to trigger the motivation to conform to the demonstrated behavior, without the need for an active joint action partner to enhance this effect.

This interpretation of our results carries practical implications for practitioners and caregivers. For example, recognizing that social motivations partly drive overimitation can inform early interventions that foster a sense of belonging and encourage prosocial or normative behaviors through group or collaborative activities. On the other hand, educators and caregivers can use this knowledge to create learning environments that balance prosocial imitation with critical thinking, for example, by encouraging children to question others’ behavior and the necessity of their actions for learning.

Finally, given the highly social nature of imitative learning contexts, we suspect that a more effective approach to isolating the effect of joint actions on imitative learning would involve a scenario where the child, as a third-party observer, watches from a distance (e.g., out of the models’ view) as two agents interact socially while jointly demonstrating an action sequence and sharing rewards at the end. The child would then be invited to manipulate the puzzle box either alone or with a partner. A benefit of such a design is that communicative cues directed at the child would be brought down to a minimum, thereby reducing the effects of an audience and the pedagogical context on learning, while still emphasizing the joint nature of the action through interactive cues and reward sharing between the models.

Results of such research could help clarify how shared goals and joint coordination inherent to joint actions guide imitation and offer practical guidance for early educators, practitioners, and caregivers in designing and implementing targeted learning interventions that account for the collaborative nature of learning.

## Figures and Tables

**Table 1:** Number of models and execution modes by experimental condition.

Condition	Number of Models Overall	Number of Models (CIA *)	Mode of Execution
Individual No Coordination	1	1	unimanually
Individual Coordination	1	1	bimanually
Joint No Coordination	2	1	unimanually
Joint Coordination	2	2	jointly (one hand each)

* Outline of the experimental conditions specifying the number of models and the coordination type (mode of execution) involved in the performance of the causally irrelevant action as well as the overall demonstration of the action sequence. Note: ‘Number of Models Overall’ refers to the number of individuals involved in the demonstration of the overall action sequence including the relevant action steps, while ‘Number of Models (CIA)’ refers to the number of individuals involved in the demonstration of the causally irrelevant action.

**Table 2:** Model summary of the logistic regression.

Model	Deviance	AIC	BIC	df	Χ^2^	*p*	McFadden R^2^	Nagelkerke R^2^	Tjur R^2^		Cox and Snell R^2^
H_0_	139.530	141.530	144.145	100							
H_1_	138.701	144.701	152.546	98	0.829	0.661	0.006	0.011	0.008		0.008
Coefficients											
					Wald Test			95% Confidence interval (odds ratio scale)			
	Estimate	Standard Error	Odds Ratio	z	Wald Statistic	df	*p*	Lower bound		Upper bound	
(Intercept)	−0.059	0.342	0.943	−0.171	0.029	1	0.864	0.482		1.844	
Coordination (low)	0.039	0.401	1.040	0.098	0.010	1	0.922	0.474		2.281	
Condition (solo)	0.362	0.401	1.436	0.902	0.814	1	0.367	0.655		3.149	

## Data Availability

Processed data supporting the results of the research study are available at: https://osf.io/hsg2n/ (accessed on 9 February 2025).

## Notes

1 Model 2 was also present in the room to ensure equivalence across conditions.

## Supplementary Materials

The following supporting information can be downloaded at https://osf.io/hsg2n/ (accessed on 9 February 2025): Processed data: Final_dataset_OI_joint_action_Rizvanovic(annonymised); Videos of the experimental demonstrations: BOX_Joint_coordination, BOX_Joint_no_coordination, BOX_Solo_coordination, BOX_Solo_no_coordination.

## Abbreviations

The following abbreviation is used in this manuscript:

CIA	Causally Irrelevant Action

## Appendix A

The following section outlines the results of exploratory analyses examining the relationship between overimitation and age, which has not been discussed in the main text.

To determine whether age was a significant predictor of overimitation in children, a logistic regression analysis was conducted, with social condition (individual vs. joint) and action coordination (yes vs. no) as fixed factors and age as a covariate. The analysis revealed no significant effect of age on overimitation rates, *p* = 0.221. The corresponding effect size, Cohen’s *d* = 0.17, indicates a small effect.

A closer analysis of the numerical differences indicates a gradual increase in overimitation rates with age, aligning with previous research and underscoring the rising importance of high-fidelity copying as a key learning strategy in childhood (McGuigan et al., 2011; Speidel et al., 2021; Whiten et al., 2016).

While the numerical increase in overimitation rates holds true for 3-year-olds (25% imitated), 4-year-olds (48% imitated), and 5-year-olds (68% imitated), there is a slight decrease observed in 6-year-olds, with 47% imitating the causally irrelevant action. However, when looking at the percentage of overimitation for the different conditions separately, the data indicate that the observed numerical decrease in overimitation in 6-year-olds might be due to the overall lower rates of overimitation for the no coordination compared to coordination conditions in this age group. For details on the four age groups, see Table A1.

**Table A1:** Overimitation rates by age group, social condition, and action coordination.

Age Group	Individual NC	Individual C	Joint NC	Joint C
3-year-olds	25% (n = 4)	0% (n = 2)	0% (n = 2)	100% (n = 1)
4-year-olds	83.33% (n = 6)	40% (n = 10)	33.33% (n = 9)	42.86% (n = 7)
5-year-olds	75% (n = 12)	62.5% (n = 8)	63.63% (n = 11)	57.14% (n = 14)
6-year-olds	33.33% (n = 3)	80% (n = 5)	20% (n = 5)	50% (n = 4)^2^
